# Supplementary material for: Association between Advanced Glycation End-Products and Sarcopenia in Patients with Chronic Kidney Disease
Source: Biomedicines. 2022 Jun 23;10(7):1489. doi: 10.3390/biomedicines10071489 (PMC9313160; doi:10.3390/biomedicines10071489)
Supplement: Supplementary file 1 [file biomedicines-10-01489-s001.zip › biomedicines-1754473-supplementary.pdf]

Table S1. Comparison between Diabetes and AGE-related parameters

Variables	Non-Diabetic	Diabetic	*p*
AGEs (arbitrary unit)	3152±829	2917±769	0,117
sRAGE (pg/mL)	2456±1289	2272±1263	0,440
esRAGE (pg/ml)	698±435	644±533	0,558
cRAGE (pg/ml)	1758±914	1642±938	0,504
AGEs/sRAGE (arbitrary unit)	1,71±1.13	1.6±0.8	0,532

AGEs, Advanced Glycation End products; sRAGE, soluble receptor for AGE; esRAGE: endogenous secretory receptor for AGE; cRAGE: cleaved receptor for AGE.
